# Self-nanoemulsifying System Optimization for Higher Terconazole Solubilization and Non-Irritant Ocular Administration

**DOI:** 10.34172/apb.2020.047

**Published:** 2020-05-11

**Authors:** Carol Yousry, Pakinam Mohsen Zikry, Emad Basalious Basalious, Omaima Naem El-Gazayerly

**Affiliations:** ^1^Department of Pharmaceutics and Industrial Pharmacy, Faculty of Pharmacy, Cairo University, Kasr El-Aini, Cairo 11562, Egypt.; ^2^Department of Pharmaceutics, Faculty of Pharmacy, October University for Modern Sciences and Arts (MSA), Egypt.

**Keywords:** Chorioallantoic membrane, High pressure liquid chromatography, Oils, Surface-active agent, Terconazole, Transmission electron microscopy

## Abstract

***Purpose:*** Eye drops’ formulations of poorly water-soluble drugs, offer the advantage of crossing the lipophilic cornea, but their limited aqueous solubility may lead to low ocular bioavailability limiting their therapeutic uses. Terconazole (TZ) is an antifungal drug with low aqueous solubility, restricting its application in ocular fungal infection. Thus, the aim of the work in this study is to enhance TZ solubilization, permitting better ocular permeation and higher bioavailability. To achieve this goal, different self-nanoemulsifying systems (SNESs) were prepared using different oils, surfactants and co-surfactants.

***Methods:*** Ternary phase diagrams were constructed to identify self nano-emulsification regions for each oil system examined; either Labrafil^®^ M2125CS or Capryol^™^ 90. TZ saturated solubility in the different formulated systems were measured and systems showing highest potential for TZ solubilization were selected. The optimized systems were chosen based on their globule size, polydispersity index, self-emulsification characteristics. Finally, TZ release as well as the irritation effect via Hen’s Egg test-chorioallantoic membrane (HET-CAM test) of the optimized system was observed in vitro.

***Results:*** The optimized system was formulated using 20% w/w Labrafil^®^ M2125 CS, 50% w/w Tween^®^ 80 and 30% w/w Transcutol^®^ HP. Oil globules showed size range of 15.13 nm and self-emulsification time of 12.80 seconds. The system released 100% of the drug within half an hour compared to 2 hours in case of TZ-suspension. Finally, HET-CAM test showed non-irritating response and normal vascularization of the chorioallantoic membrane.

***Conclusion:*** The formulated SNES could be a promising approach to enhance ocular efficacy of TZ.

## Introduction

The high incidence rate of fungal infections and the slow progress in developing new antifungal molecules,^1^ forced the pharmaceutical market to propose novel drug systems with better delivery approaches; either topically or orally.^2+,3^ The major requirement for such delivery systems is enhanced antifungal bioavailability with reduced toxicity.^4^

For ocular fungal treatment, drug formulations are usually available as eye drops, whether solutions or suspensions.^5^ These are usually poorly absorbed; due to corneal impermeability and short residence time in the ocular mucosa.^6^ This short residence time of eye drops, is attributed to the low-volume capacity of precorneal area. The volume of lacrimal lake and pool of tears in the cul-de-sac ranges between 7 and 10 µL and it can accommodate up to 30 µL maximum. However, the average eye drop size (almost 50 µL) exceeds the ocular capacity leading to the loss of around 40% of the instilled volume.^7^ Thus, frequent applications of eye drops are required to overcome this problem and meet the minimum therapeutic efficacy. Unfortunately, frequent instillations lead to nasolacrimal absorption causing systemic side effects and patient incompliance.^8^

Eye drops of poorly water-soluble drugs, offer the advantage of crossing the lipophilic cornea, but their low aqueous solubility may lead to low ocular bioavailability limiting their therapeutic uses. Many efforts are exerted to enhance the ocular bioavailability by improving drug solubility and increasing their ocular retention which subsequently may result in better ocular absorption.^9^

Terconazole (TZ), a broad spectrum antifungal agent that acts through inhibition of the fungal cytochrome P-450.^10^ TZ is a poorly water-soluble antifungal drug; that suffers low ocular absorption which limits its ocular application despite its good efficacy. Several approaches were utilized to incorporate poorly water-soluble drugs into novel drug delivery systems to enhance their clinical potential. Loading such drug molecules into inert lipid vehicles in a nano-metric range may improve their dissolution rate and maximize their bioavailability.^11-13^ Thus, self-nanoemulsifying systems (SNESs) may offer great improvement in the *in vitro* and *in vivo* performance of poorly water-soluble drugs and therefore, may serve as good vehicles for ocular drug delivery.

SNES provides a homogeneous, transparent and stable liquid formulation.^14^ It is an anhydrous type of emulsion: consisting of a combination of oil, surfactant, co-surfactant and drug which is thermodynamically stable.^4^ Then the mixture forms a fine o/w nanoemulsion upon gentle agitation with the aqueous phase. SNES provides low interfacial tension and large o/w interfacial areas, which offers higher solubilization abilities, leading to better incorporation of poorly water soluble drug inside the oil droplets resulting in higher drug permeation.^15^ They also have longer shelf life and higher drug stability.^4^ The type of oil, surfactant and co-surfactant used in SNES formulation depend on the solubility of the drug in different media, surfactant/co-surfactant emulsifying capacity, zone of self-emulsification and the extent of droplet size distribution in the nanoemulsion.^16^

Based on these considerations, the aim of our study is to develop, optimize, characterize and evaluate TZ- loaded SNES in order to enhance solubilization capacity of this poorly water-soluble drug for proper treatment of ocular fungal infections. Phase solubility of TZ was evaluated in various oils, surfactants, and co-surfactants. Pseudo ternary phase diagrams were constructed to identify the optimized self-nanoemulsification areas. The formulations were evaluated by measuring globule size, polydispersity index, self-emulsification time, *in vitro* precipitation test, *in vitro* release study and finally, Hen’s egg test-chorioallantoic membrane (HET-CAM) assay was performed to assess the irritation of the prepared system.

## Materials and Methods

### Materials 

Terconazole was supplied as a gift from Marcyrl Pharmaceutical Industries (Cairo, Egypt). Labrafil^®^M2125 CS, Capryol^™^90, Labrasol^®^, Transcutol^®^ HP, Lauroglycol^™^90 and Maisine^™^35-1 were kindly donated by Gattefosse (Lyon, France). Miglyol^®^812 was donated from Cremer Oleo division (Hamburg, Germany). Cremophor^®^RH40 was donated by BASF (Ludwigshafen, Germany). Captex^®^8000 was a gift from Abitec Corporation, Janesville. Tween^®^80, methanol, ethanol, sodium hydroxide, sodium dihydrogen phosphate, sodium lauryl sulphate, glycerin and propylene glycol were obtained from Adwic, El-Nasr pharmaceutical chemicals Co. (Cairo, Egypt). All reagents and solvents used in this study were of analytical grade.

### Determination of TZ solubility in oils, surfactants and co-surfactant

Different oils were selected to create diverse emulsifying regions.^4^ TZ solubility was tested in different oils namely; Labrafil^®^ M 2125 CS, Miglyol^®^ 812, Capryol^™^90, Maisine^™^ 35-1 and Captex^®^8000, different surfactants; Labrasol^®^, Cremophor^®^ RH40 and Tween^®^80, as well as different co-surfactants/ co-solvents; Lauroglycol^™^ 90, propylene glycol, Transcutol^®^ HP, glycerin and ethanol. Excess amount of TZ was added to 4 mL of each studied vehicle followed by vortex mixing for 5 minutes. Vials were kept in a water bath shaker at 37°C for about 48 hours. After reaching equilibrium, the mixtures were centrifuged at 4000 rpm for about 15 minutes and the supernatant was collected, diluted with methanol, and measured spectrophotometrically at λ_max_ of 296 nm. All systems were prepared in duplicate.

### Construction of pseudo-ternary phase diagrams using the selected oils/surfactant/co-surfactant

**Figure 1:**
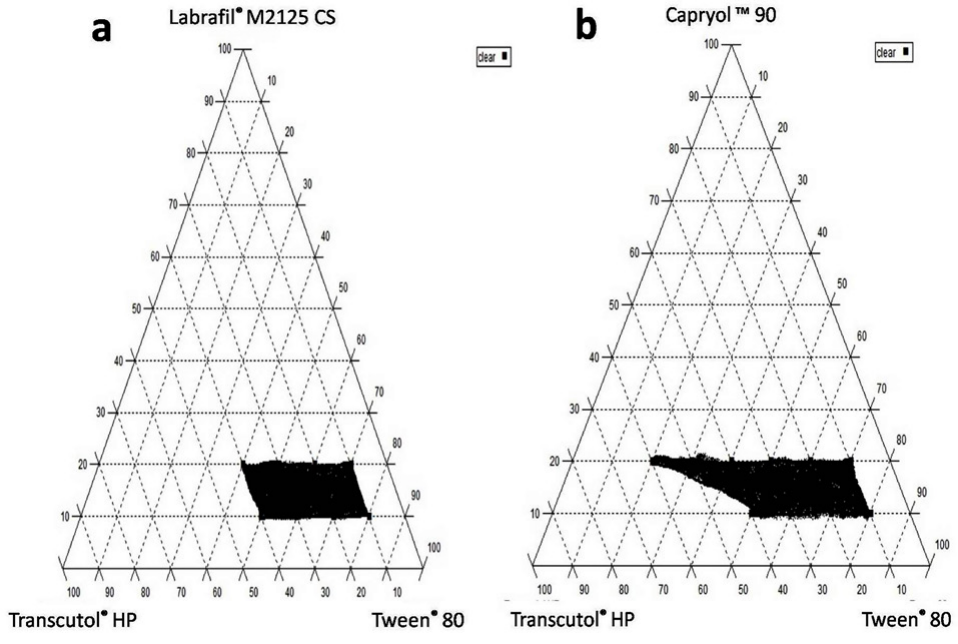
Pseudo-ternary phase diagram showing the self nanoemulsifying region for Labrafil® M 2125 CS-based systems (a) and capryol™ 90 -based systems (b).

SNES forms fine o/w emulsion when present in aqueous media just by simple agitation. Surfactant and co-surfactant/co-solvent reduce interfacial tension and provide a mechanical barrier preventing the globules from coalescence, by preferring adsorption at the interface. Based on the results of the solubility study, the selected oils (Labrafil^®^M 2125 CS and Capryol^™^90), surfactant (Tween^®^ 80) and co-surfactant/co-solvent (Transcutol^®^ HP) were chosen to construct pseudo-ternary phase diagrams, using distilled water as the aqueous phase, to obtain the range in which self-nanoemulsifying region exists.^17^ The concentrations of oils, surfactant and co-surfactant/co-solvent varied between 10 and 20% (w/w), 20 to 80% (w/w) and 10 to 60% (w/w) respectively. The total of surfactant, co-solvent and oil concentrations, for any mixture, always added to 100%.^18^ Ternary mixtures with varying percentages of components were prepared, resulting in a total amount of 0.1 g to which 20 mL of distilled water was added. Two different pseudo-ternary phase diagrams of surfactant, co-solvent and oils (without incorporation of the drug) were plotted, each representing a side of the triangle (one diagram for each oil; Figure 1). The formation of the nanoemulsion was visually observed as clear/transparent, easily dispersible with low viscosity.^19^ Mixtures appearing turbid, viscous or separated in phases were rejected. The phase diagrams were drawn using CHEMIX ternary plot software (CHEMIX School ver. 3.60, Pub. Arne Standnes, Bergen, Norway).

### Preparation of TZ saturated SNES and determination of solubilization capacity 

Once pseudo-ternary diagrams identified the self-emulsifying region, eighteen different systems were prepared as shown in Table 1.^20^ Excess TZ was dissolved in the previously prepared systems and mixed continuously for 2 minutes using vortex mixer (Stuart SA8; UK). The prepared TZ- loaded SNES were kept in tightly closed glass vials in a water bath shaker at 37°C for 48 hours. After reaching equilibrium, the different systems were centrifuged at 4000 rpm for about 15 minutes. Finally, 20 mL distilled water was added to 0.1 g of the supernatant to detect its visual clarity.

**Table 1:** Composition of different self nanoemulsifying systems with different oils:surfactant:co-surfactant ratios and their measured dependent responses

**System**	**Labrafil** ^®^ **M** **Oily phase** **(%; w/w)**	**Capryol ™ 90** **Oily phase** **(%; w/w)**	**Tween** ^®^ **80** **Surfactant** **(%; w/w)**	**Transcutol** ^®^ **HP** **Co-surfactant** **(%; w/w)**	**Terconazole solubilty** **(mg/mL)**	**Globule size** **(nm)**	**PDI**	**Self-emulsification time (s)**
SNES L1	10		80	10	26.00	-	-	-
SNES L2	10		70	20	42.60	-	-	-
SNES L3	10		60	30	50.25	-	-	-
SNES L4	10		50	40	57.00	-	-	-
SNES L5	20		70	10	-	-	-	-
SNES L6	20		60	20	42.40	-	-	-
SNES L7	20		50	30	72.70	15.13 ± 0.34	0.07 ± 0.01	12.80
SNES L8	20		40	40	71.00	20.26 ± 0.08	0.10 ± 0.01	14.34
SNES C1		10	80	10	36.00	-	-	-
SNES C2		10	70	20	39.93	-	-	-
SNES C3		10	60	30	54.25	-	-	-
SNES C4		10	50	40	58.50	-	-	-
SNES C5		20	70	10	47.80	-	-	-
SNES C6		20	60	20	61.90	59.87 ± 0.16	0.12 ± 0.02	29.82
SNES C7		20	50	30	72.50	59.00 ± 1.36	0.09 ± 0.01	31.13
SNES C8		20	40	40	-	-	-	-
SNES C9		20	30	50	-	-	-	-
SNES C10		20	20	60	-	-	-	-

PDI, poly dispersity index

Based on their optical clarity, 14 systems namely (SNES L1- SNES L4, SNES L6- SNES L8 and SNES C1-SNES C7) were selected. For these selected systems, another portion of the supernatant was collected and diluted with methanol to determine TZ concentration spectrophotometrically at λ_max_ of 296 nm. All systems were prepared and characterized in duplicate.

### Characterization of the selected TZ- loaded SNES

Four systems (SNES L7, SNES L8, SNES C6 and SNES C7) showing the highest oil content (20%) and highest drug solubility; were selected from the previously prepared systems for further evaluation and characterization.

### Determination of globule size and polydispersity index (PDI) for the selected SNES

Accurately 0.1 mL TZ-loaded SNES (10 mg drug/mL system) was diluted with distilled water (1:100) then mixed for 1 min using vortex mixer. The globule size and PDI were measured using Zetasizer Nano ZS (Malvern Instruments, Malvern, UK). A Helium–Neon (He-Ne) laser beam at 632 nm wavelength was used and light scattering was monitored at 25°C.

### Assessment of self-emulsification time and in vitro precipitation test

Based on the solubility studies and pseudo-ternary phase diagrams, the four selected SNES systems were prepared. The self-emulsification property of the prepared SNES was evaluated by assessing its emulsification time and drug precipitation.^21,22^ One mL of each of the formulated TZ-loaded SNES (10 mg TZ/mL) was mixed with 200 mL phosphate buffer pH 7.4 maintained at 37°C using magnetic stirrer. The self-emulsification behavior was visually observed and the time needed for complete emulsification of the SNES was recorded.

For the *in vitro* precipitation test; one mL of each of TZ-loaded SNES was added to 200 mL of phosphate buffer pH 7.4 at 37°C, and agitated gently using magnetic stirrer. Samples, each of 3 mL; were withdrawn from the phosphate buffer medium without volume replenishment at different time points (1, 5, 15, 30, 60 and 120 minutes). These samples were then analyzed for drug content measurement using high performance liquid chromatography (Waters Alliance HPLC system, Milford, Massachusetts, USA) installed with Zorbax eclipse C_18_ column (250 x 4.6 mm, 5 µm). A 100 µL sample was injected at 45°C where TZ was eluted using Acetonitrile: 0.01M ammonium acetate buffer (55:45, v:v) as mobile phase at flow rate of 1.5 mL/min and measured at wave length 220 nm. Finally, the average percentage of the TZ was plotted against time. All experiments were conducted in duplicate.

### TZ-loaded SNES optimization

Based on the previous evaluation, the system showing the smallest globule size, least PDI, shortest self-emulsification time and highest resistance to precipitation; was selected as the optimized system for further characterization.

### Characterization and evaluation of the optimized system Transmission electron microscopy (TEM)

The dispersed oil droplets of the optimized system (SNES L7) were examined morphologically by transmission electron microscopy (TEM) which was operating at 80 kV (model JEM-1230, Jeol, Tokyo, Japan). One drop of the diluted system was placed on a carbon coated copper grid and left to dry in air for 10 minutes. Afterwards, the sample was negatively stained with 2% Phosphotungstic acid solution for 60 seconds and excess solution was removed using filter paper. The system morphology and surface topography were observed at appropriate magnifications.

### In vitro TZ release study

The release profile of TZ from the optimized SNES L7 was performed using a USP dissolution tester (Apparatus II; paddle method) with some modifications.^23^ Two milliliters of the optimized system (containing 2 mg TZ) was added into 250 mL phosphate buffer pH 7.4 containing 1% sodium lauryl sulphate (SLS) at 37°C. The paddle was rotated at 50 rpm and samples of 3 mL were withdrawn at predetermined time intervals (0.25, 0.5, 1, 2, 4, 6, and 8 hours) with volume replacement to maintain a constant volume of release medium. Samples were filtered through 0.45µm pore sized nylon syringe filter, and the filtrates were subsequently analyzed using HPLC to determine the amount of TZ released. TZ release from drug suspension (2 mg/2 mL) was performed for comparative evaluation. All experiments were performed in duplicate.

### Stability studies of the optimized system

The zeta potential of the formulated optimized SNES L7 was evaluated using Zetasizer Nano ZS and its physical stability was assessed as follows:

### Thermodynamic stability study

SNES is considered a highly stable emulsified system that resist precipitation, creaming or cracking upon exposure to extreme conditions of temperature and centrifugal force. Thus, the thermodyanmic stability of the optimized system was assessed as following^20^:

Centrifugation study: The optimized SNES L7 was centrifuged (Heraeus Megafuge 16R; ThermoFisher scientific, Germany) at 5000 rpm for 30 minutes and the system was visually inspected for any signs of precipitation, creaming, craking or phase separation.

Heating and cooling cycle: The optimized SNES L7 was subjected to three cycles of cooling and heating at 4°C and 45°C respectively with storage at each temperature for 24 hours. The system was visually inspected as before.

Freeze thaw cycle: This test involved three freeze-thaw cycles at temperatures between -20°C and 25°C with storage for 24 hours at each temperature. The system was visually inspected for any signs of phase separation, precipitation, creaming or cracking.

### Shelf stability study

Shelf stability of the optimized TZ-loaded SNES L7 was performed by keeping the system into sealed glass vials at ambient room temperature (25-30°C) for 6 months. The physical stability of the system was evaluated by comparing the globule size and zeta potential values of the system before and after the 6 months storage. In addition, visual inspection of the system for drug precipitation, phase separation or creaming was performed.^20^

### In vitro irritation test: Hen’s egg test-chorioallantoic membrane (HET-CAM)

**Figure 2:**
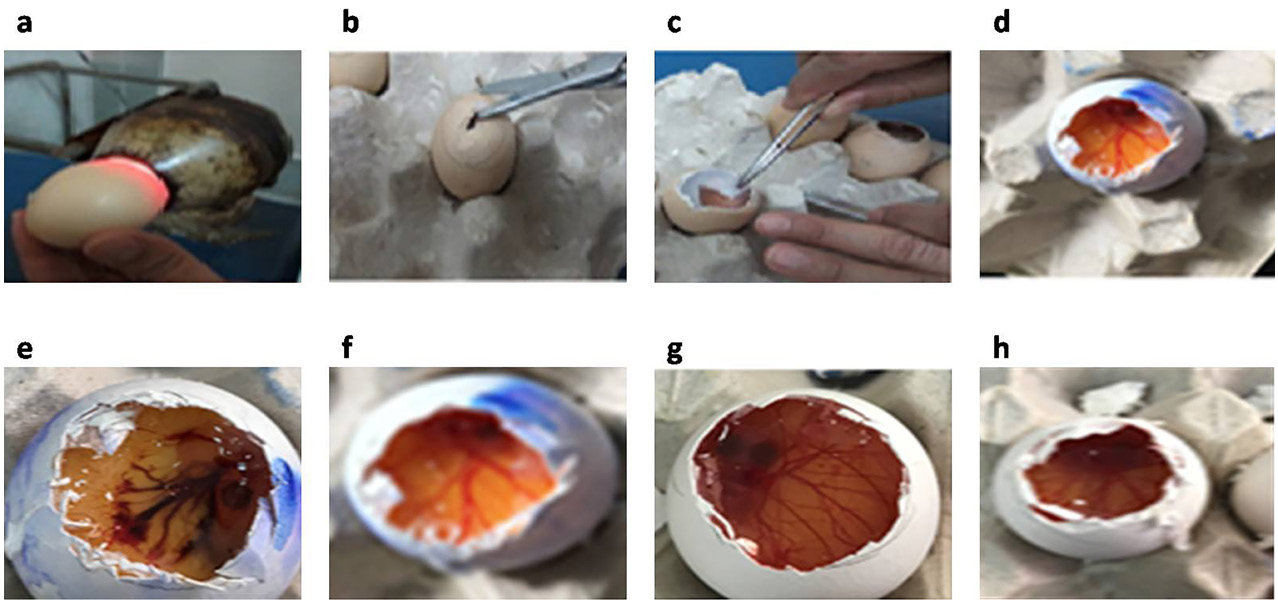
Diagrammatic scheme showing the procedural steps for preparing the Hen’s egg test-chorioallantoic membrane (HET-CAM) test (a-d); where the egg was candled to check the embryos-viability and to mark the air space (a), the external shell was removed (b), the inner membrane was removed (c) and the normal vascularization of the chorioallantoic membrane was shown (d) as well as the observed responses to different systems application (e-h); the positive control (10% NaOH; e), the negative control (0.9% NaCl; f), Terconazole-loaded SNES L7 (g) and Terconazole-suspension (h).

Fresh fertilized Hen’s eggs were incubated at 37.5 ± 0.5°C and 66 ± 5 % relative humidity for 10 days. Eggs were rotated on each side three times daily to avoid sticking of the embryo to one side of the egg. After ten days, the eggs were candled to get sure of the viability of the embryos and to mark the airspace using a pencil (Figure 2a). Defective eggs were discarded. The egg shell around the air space was removed finely using blunt forceps and a scissors (Figure 2b). After the exposure of the inner membrane, it was sprayed with 0.9% NaCl solution to keep it warm and moist till use. Immediately prior to use, the inner membrane was removed using a forceps (Figure 2c) to reveal the chorioallantoic membrane (CAM) (Figure 2d).^24^ The tested substances (the optimized system (SNES L7) and TZ-suspension), in a volume of 0.3ml, were applied directly to the CAM, covering 50% of its surface for 5 minutes.^25^ Negative control (0.9% NaCl aqueous solution) and positive control (10% NaOH aqueous solution) were also applied on other eggs for comparison. The test was performed in triplicate.

After treatment of CAM, they were examined for any vascular changes, such as, hyperemia (i.e. congestion due to the increment in blood flow), hemorrhage (i.e. red spots around the vessels due to its bleeding), lysis (i.e. contraction or disappearance of the vessels due to vessel spasm), clotting and coagulation (detected by dark spots: around (extra vascular coagulation) or inside (intravascular thrombosis) the vessels, or coagulation of albumen which appeared as a white color on the CAM.^26^ Occurrence of any of these changes to the CAM reflects the potential tendency of the test compound to damage the mucous membrane especially; the eye when applied *in vivo*.

## Results and Discussion

### Determination of TZ solubility in oils/ surfactants/co-surfactant

**Figure 3:**
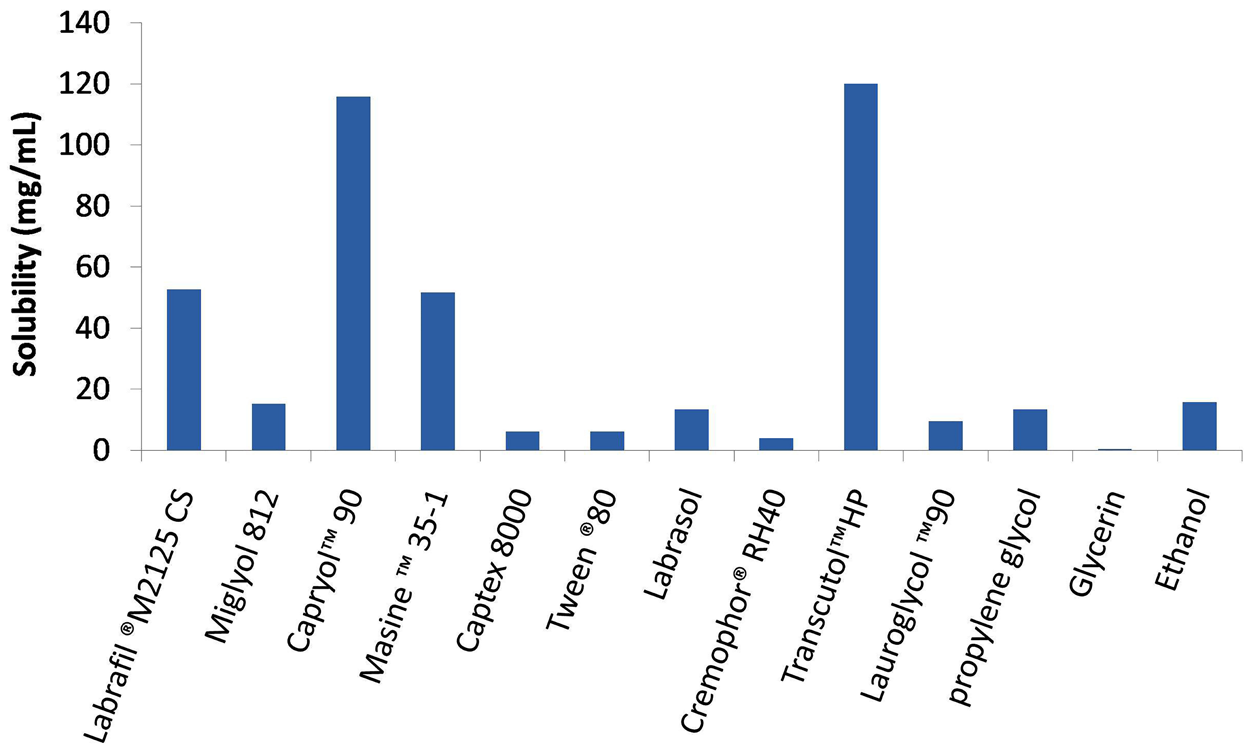
Saturated solubility of terconazole in different oils, surfactants and co-surfacatants to select the optimum self-nanoemulsifying components.

SNES behaves as monophasic liquid system consisting of oil, surfactant and co-surfactant/co-solvent loaded with the drug under investigation.^21^ Therefore, proper selection of its components is the key factor to fulfill the highest drug loading, solubility and stability of the system.^27^ Terconazole saturated solubility in various vehicles: oils, surfactants and co-surfactants are illustrated in Figure 3. Oils with highest potential of drug solubility should be typically selected as the oil phase of SNES for its major role in establishing maximum drug loading in the formulation.^28^ Amongst the five oils studied, Capryol^™^90 exhibited the highest drug solubility reaching 115.63 mg/mL, therefore it was chosen as the oily phase. Capryol^™^90, a saturated medium chain triglyceride (eight carbon) with an amphiphilic nature, impart both self-emulsification and surfactant properties which led to its high efficiency in SNES formulation.^29^ Also, Labrafil^®^ M 2125 CS, a mixture of mono-, di- and triglycerides and PEG-6 mono- and diesters of linoleic acid; showed the second highest TZ solubility (52.66 mg/mL). Labrafil^®^ M2125CS has the ability to self-emulsify on contact with aqueous media and a fine nanoemulsion may be formed upon its combination with other suitable surfactants. Thus, it can function as a lipid vehicle for poorly water soluble drugs.^30^

Among the screened surfactants, Labrasol^®^ and Tween^®^ 80 were also selected due to their highest drug solubility (13.38 and 6 mg/mL respectively). Generally, non-ionic hydrophilic surfactants with proper hydrophilic/lipophilic balance value (HLB) are of great value because of their detected stability.^31^ They are usually used in nanoemulsion formulations and pharmaceutical preparations as they are less affected by pH and ionic strength changes,^32^ have lower critical micelle concentration compared to their ionic counterparts,^33^ and usually resulting in a less toxic and irritant formulations.^34^ In addition, Tween^®^80 and Labrasol^®^(HLB >10), have shown proven superiority in forming uniform small emulsion droplets,^9^ thus allowing rapid drug release and absorption.^35^ These two surfactants were reported to have bioenhancing activity; where Labrasol^®^ affects the tight junction between cells enhancing paracellular transport and Tween^®^ 80 has shown an inhibitory effects on P-glycoprotein and cytochrome P450 enzymes.^34^ The choice of co-surfactant/co-solvent is as important as other ingredients in SNES formulations as it allows more drug loading, equilibrate self-nanoemulsification time and optimize the globule size, in addition to widening the area of self-nanoemulsification in the pseudo-ternary phase diagrams.^28^ In a nanoemulsion, co-surfactant decreases interfacial tension between the oil and aqueous phases by increasing the film flexibility and curvature so augmenting the spontaneous self-emulsification action of the used surfactant.^35^ Transcutol^®^ HP was selected as co-surfactant/co-solvent owing to its highest drug solubilization power (120 mg/mL). Its good solubilization effect ensures high drug loading capacity in addition to its absorption and permeability enhancement power.^36^

### Construction of pseudo-ternary phase diagrams using the selected oils/surfactant/co-surfactant

Thirty-six different systems were formulated using different oil: surfactant: and co-surfactant/co-solvent combinations. Based on the visual observation, Labrasol^®^ was excluded from the design because it failed to produce any transparent SNES. On the other hand, 18 different systems (Table 1) combining; Labrafil^®^M2125 CS or Capryol^™^90 as the oily phase with Tween^®^ 80 and Transcutol^®^HP were found clear and maintained their clear physical appearance for 24 hours post-dilution confirming the stability of the nanoemulsions.^37^ Pseudo-ternary phase diagrams; presented as shaded areas in the phase diagrams (Figure 1); were constructed in the absence of TZ to identify the self-nanoemulsifying regions of each system separately.^23^

### Preparation of TZ saturated SNES and determination of solubilization capacity

The drug-loaded SNES should appear clear, as a monophasic liquid at room temperature, and must have good solvent properties upon its introduction to aqueous phase.^35^ Thus, after TZ incorporation into the previous eighteen systems, only fourteen systems remained clear and were measured spectrophotometrically to detect the maximum drug solubility. As shown in Table 1, TZ-solubility in the different SNES ranged from 26 to 72.7 mg/mL compared to TZ solubility of 0.0116 mg/mL in distilled water. These results fulfill the main need for SNES formulation to enhance drug solubilization and permeation.

### Characterization of the selected TZ- loaded SNES

Based on the results of drug solubility and the oil content of the different systems, four systems namely; SNES L7, SNES L8, SNES C6 and SNES C7, were selected for further investigations to optimize the results and prepare the most stable and efficient system. Drug content of these different systems was 10 mg TZ/mL.

### Determination of globule size and PDI for the selected SNES

Desirable stable SNES must show small globule size and small PDI in diluted measurements.^20^ Globule size analysis is a critical parameter in evaluating the drug delivery performance and stability of SNES. Where small globule size (<100 nm) leads to larger interfacial surface area, leading to higher drug release from the SNES and consequently, better drug absorption.^38,39^ In our study, the globule size of the selected systems ranged from 15.13 to 59.87 nm as shown in Table 1 fulfilling the SNES criteria.^40^ Tween^®^ 80 as a surfactant, promoted the formation of reduced size globules by improving the flexibility of the oil/water interface.^41^ The relative percentage of surfactant/co-surfactant affects the globule size by different ways.^42^ Increasing surfactant percentage resulted in smaller globule size due to localization of the surfactant molecules at the oil/water interface causing stabilization of the interfacial film which subsequently lead to stabilization of the oil droplets. On the contrary, increasing co-surfactant percentage may cause expansion of the interfacial film^27,43^; which may lead to globule size increment. Therefore, surfactant/co-surfactant percentage must be properly balanced.

SNES formulated using Labrafil^®^M2125 as oily phase showed smaller globule size when compared to those formulated using Capryol^™^90. This could be attributed to the higher emulsifying ability of Labrafil^®^M2125 CS and its hydrophilic/lipophilic balance which reduce the water-oil interfacial tension leading to smaller globule size.^44^

The PDI is used to assess the nanoemulsion homogeneity, i.e. the globule size distribution in the systems.^45^ The PDI of all the formulated systems shown a small PDI ranging from 0.07 to 0.12 indicating high degree of globule size uniformity.^19^

### Assessment of self-emulsification time and in vitro precipitation test

Poorly soluble drugs usually suffer high chance of precipitation upon system dilution. Therefore, self-emulsification time and precipitation evaluation are critical to assess the stability of the drug-loaded system to overcome such problem.^39^

The emulsification time is considered an important index of the efficiency of formula undergoing self-emulsification.^46^ A successful SNES should emulsify completely and quickly in aqueous medium.^21^ All the selected systems (SNES L7, SNES L8, SNES C6 and SNES C7) exhibited a rapid emulsification time, which ranged from 12.80 to 31.13 seconds (Table 1). This rapid emulsification time could be attributed mainly to the use of Tween^®^ 80 as a surfactant in the four systems which resulted in high emulsification efficiency and short emulsification time.^37^

**Figure 4:**
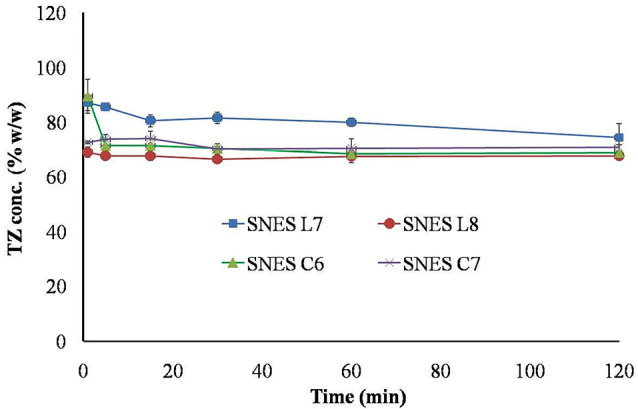
In vitro terconazole precipitation after dilution of different TZ-loaded SNES.

On the other hand, the *in vitro* precipitation test, must be conducted under non-sink conditions in order to recognize the tendency of the drug in SNES to precipitate when subjected to aqueous medium.^47^ When drug-loaded SNES are subjected to aqueous medium, the drug might exist as solubilized globules borne inside the emulsion or as precipitated form of the drug. Therefore, samples were filtered to remove the precipitated TZ before drug concentration in the solubilized globules was measured. The results for the *in vitro* precipitation test are demonstrated in Figure 4. All the examined systems showed high resistance to drug precipitation and retained most of its solubilized TZ level constant for the experiment time (120 minutes).

### Characterization and evaluation of the optimized system

TZ-loaded SNES (SNES L7) was selected as the optimized system of SNES as it shows the smallest globule size (15.13 nm), least PDI (0.07), shortest emulsification time (12.80 seconds) and highest resistance to drug precipitation. SNES L7 was formulated using 20% w/w Labrafil^®^M2125 CS, 50% w/w Tween^®^ 80 and 30% w/w Transcutol^®^HP.

### Transmission electron microscopy (TEM)

**Figure 5:**
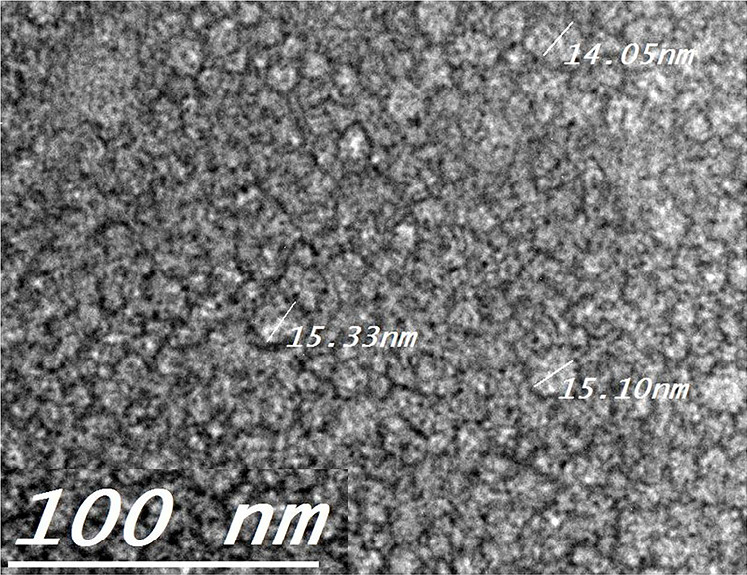
Transmission electron microscopy (TEM) showing the morphology and the globule size of the optimized Terconazole-loaded SNES L7

The TEM of the optimized SNES L7 shown in Figure 5 revealed dispersed spherical oil globules with size range of 16 nm as recorded by the globule size measurement by Zetasizer.^37^ The image shows a thick monolayer of surfactant/co-surfactant surrounding each globule. This layer forms a barrier against globules coalescence which leads to a stable nanoemulsion formulation.^48^ In addition, the TEM did not show any precipitated drug which may interfere with the physical stability of the formed nanoemulsion.^27^

### In vitro TZ release study

**Figure 6:**
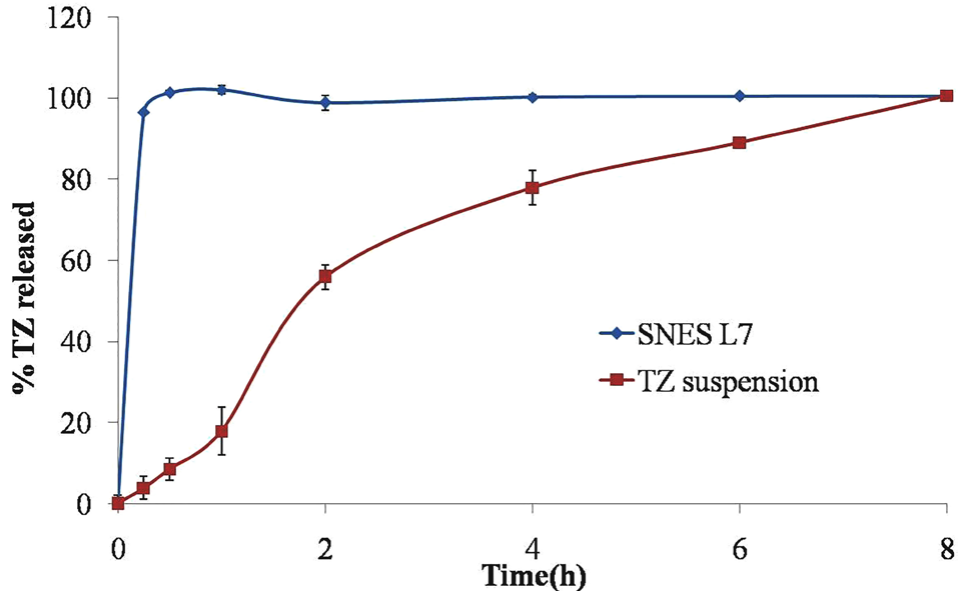
In vitro release profiles showing Terconazole release from the optimized Terconazole-loaded SNES L7 and Terconazole suspension.

The study of TZ release from the prepared SNES is important to predict its *in vivo* behavior and ensure the drug release and absorption.^39,49^ It could be observed from Figure 6 that TZ release from the optimized SNES L7 was extremely fast when compared to TZ suspension where 100 % w/w of TZ was released within half an hour in the SNES compared to 8 hours for TZ suspension. This fast release behavior could be attributed mainly to TZ solubilization, the small globule size of the emulsion, its low PDI and rapid emulsification time. Upon dilution with water, the SNES L7 composed of oil, surfactant and co-surfactant; is rapidly emulsified forming small globules with large interfacial surface area, resulting in rapid TZ solubilization and high drug release.^50^

This may assure the complete release of the drug from the system before its drainage and loss from the ocular tissue which will subsequently result in higher TZ ocular permeation.

### Stability studies of the optimized system

The zeta potential value of the optimized SNES L7 was measured and found to be -13.00 ±1.01 mV. Thus, the thermodynamic stability and shelf stability study was performed to ensure the physical stability of the formulation.

### Thermodynamic stability study

The thermodynamic stability of the optimized SNES L7 was assessed by visually observing its resistance to phase separation, creaming or any anticipated changes upon exposure to extreme conditions of temperature and centrifugal forces. The optimized system maintained its integrity and didn’t show any signs of precipitation, creaming or cracking indicating the physical stability of the system and its high tolerance to extreme condition

### Shelf stability study

The investigated optimized SNES L7 was physically stable with no changes in its visual appearance (no signs of phase separation, drug precipitation or creaming). The globule size and zeta potential values after 6 months storage were comparable to values of the freshly prepared system (Table 2) indicating highly stable system that retains its physical stability upon storage.

**Table 2:** Globule size and zeta potential values for the optimized self nanoemulsifying system before and after 6 months storage

	**Globule size (nm)**	**Zeta Potential (mV)**
Before 6 months storage (Fresh system)	15.13 ± 0.34	-13.00 ±1.01
After 6 months storage	18.66 ± 0.05	-09.61 ± 1.12

### In vitro irritation test: HET-CAM

The HET-CAM test was performed to investigate the potential safety of the optimized system SNES L7 by observing any possible irritation that may occur to the ocular tissues upon its application. The perfect vascularization of CAM tissue (arteries, veins and capillaries) clearly shows the inflammatory reaction in response to injury in a similar way to the rabbit’s conjunctival tissue.^26^ As illustrated in Figure 2e, the positive control (10% NaOH) was severely irritant resulted in severe hyperemia and hemorrhage. On the other hand, 0.9% NaCl; which is used as negative control, produced non-irritant response in the form of normal tissue vascularization (Figure 2f). Same results were obtained with the optimized SNES L7 (Figure 2g) as well as TZ-suspension (Figure 2h) where both systems exhibited non-irritant effect on the CAM without any vascular damage. These findings could confirm the safe and non-irritant characteristics of the optimized TZ-loaded SNES as a potential platform to deliver TZ efficiently to the ocular tissue for antifungal treatment.

## Conclusion

In this study, we managed to prepare SNES to enhance TZ solubilization, release and subsequently ocular penetration. Different oils, surfactants and co-surfactants were examined for their drug solubilization and different systems were prepared and optimized to produce system showing small globule size with rapid self emulsification properties and high stability. This optimized system was formulated using 20% w/w Labrafil^®^ M2125 CS, 50% w/w Tween^®^ 80 and 30% w/w Transcutol^®^HP and showed globule size of range 15.13 nm and 12.80s emulsification time. The fast release profile of TZ from the optimized system allows its complete release from the system before its drainage from the ocular tissues. Finally, the irritant behavior of the optimized system was investigated *in vitro* using the HET-CAM test to assure its safe ocular application. Future studies to investigate *in vivo* ocular irritation, ocular permeation and efficacy of the formulated optimized system is needed.

## Ethical Issues

The use and handling of the chorioallantoic in this study were approved by the research ethical committee-Faculty of Pharmacy, Cairo university (PI(1051)).

## Conflict of Interest

The authors declare no conflict of interest.
